# Advancing Telephone Focus Groups Method Through the Use of Webinar: Methodological Reflections on Evaluating Ontario, Canada’s Healthy Babies Healthy Children Program

**DOI:** 10.1177/2333393615607840

**Published:** 2015-10-05

**Authors:** Eunice Chong, Adrienne Alayli-Goebbels, Lori Webel-Edgar, Sarah Muir, Heather Manson

**Affiliations:** 1 Public Health Ontario, Toronto, Canada; 2 University Hospital of Cologne, Germany; 3 Simcoe Muskoka District Health Unit, Barrie, Ontario, Canada; 4 University of Toronto, Ontario, Canada

**Keywords:** community and public health, focus groups, group interaction, program evaluation, research, online, technology

## Abstract

Telephone focus groups have been increasingly popular in public health research and evaluation. One of the main concerns of telephone focus groups is the lack of nonverbal cues among participants, which could limit group interactions and dynamics during the focus group discussion. To overcome this limitation, we supplemented telephone focus groups with webinar technology in a recent evaluation of a provincial public health program in Ontario, Canada. In this article, we share the methods used and our experiences in conducting telephone focus groups supplemented with webinar technology, including advantages and challenges. Our experience will inform other researchers who may consider using telephone focus groups with webinars in future research and evaluation.

Focus groups are widely used to collect qualitative data in a group setting and are traditionally conducted face-to-face. For the past decade, researchers have used other means to conduct focus group discussions, such as Internet and telephone (Fox, Morris, & Rumsey, 2007; Gothberg et al., 2013; Krueger & Casey, 2009). Telephone focus groups emerged as a method in health research to explore views and experiences of patients and health professionals regarding preventive health services, treatment options, and other topics, such as employment experiences after diagnosis of cancer (Frazier et al., 2010; Horowitz, Siriphant, Canto, & Child, 2002; Koskan et al., 2014; Ross, Stroud, Rose, & Jorgensen, 2006; Smith, Sullivan, & Baxter, 2009a). In two separate literature reviews of published health-related studies that used telephone focus groups (Cooper, Jorgensen, & Merritt, 2003; Smith et al., 2009b), the authors identified several advantages: that telephone focus groups are useful to assemble participants from geographically disparate locations and from a wide range of practice settings, can accommodate participants’ schedules, increase participant anonymity by eliminating visual contact, increase participation rates through eliminating the need for participants to travel, and reduce time and monetary costs of focus groups.

Telephone focus groups also have several limitations, such as potential sampling bias and the lack of generalizability of findings because participants are limited to those who have access to telephone (Smith et al., 2009b). In particular, the lack of nonverbal cues in telephone focus groups is commonly identified as an important limitation, because it may reduce interactions between participants and prohibit moderators from observing and acting on these interactions (Gothberg et al., 2013). Interactions are considered the most important characteristic of focus groups that differentiates them from other data collection methods (Gill, Stewart, Treasure, & Chadwick, 2008; Gronkjaer, Curtis, de Crespigny, & Delmar, 2011; Krueger & Casey, 2009). Through asking questions of each other, seeking clarifications, and commenting on the topics, participants encourage each other to reveal more information, and as the discussion progresses, the generated data become sharper and more refined (Finch & Lewis, 2003). Therefore, group interactions provide a richness of data that may not be accessible from other data collection methods (Morgan, 1997).

The challenge of reduced interaction in telephone focus groups may be overcome by recent webinar technology, which includes specific features to enhance interactions. A webinar is a computer-mediated communication system that is used to exchange information in a real time and two way format (Wang & Hsu, 2008), and is now commonly used for online learning and training through providing a virtual classroom environment to teach students or trainees. According to Wang and Hsu (2008), the webinar “provides a nearly face-to-face environment that increases participants’ social presence and facilitates multi-level interaction.” It is not meant to replace face-to-face interaction but is a collaborative technology specially designed to support and enhance human interaction and teamwork (Marjanovic, 1999).

In the scientific literature, little is known about the application of webinar technology to augment telephone focus groups for research or evaluation purposes. This article addresses this knowledge gap by sharing our experiences and reflecting on the process of using this method in a recent evaluation of the implementation of a public health program in Ontario, Canada. We highlight the advantages and challenges of using the webinar technology to supplement telephone focus groups.

## Ontario’s Healthy Babies Healthy Children Program

Ontario is the second largest province in Canada, with 36 health units serving a total population of 12,851,821 people spread across 908,608 square kilometers (Statistics Canada, 2012). Ontario is very diverse in terms of geographic (i.e., urban vs. suburban vs. remote communities) and demographic (i.e., age, income, culture) characteristics. Funded by the Ontario Ministry of Children and Youth Services (MCYS), Healthy Babies Healthy Children (HBHC) is a public health program designed to help infants and children (ages 0 to 6 years) across Ontario to have a healthy start in life and provide them with opportunities to reach their full potential (MCYS, 2011). This voluntary program is delivered through all 36 health units in partnership with hospitals and other community partners. Key program components include universal postpartum screening and targeted assessments to identify any risks that could affect child development. Public health nurses and family home visitors support vulnerable families by providing evidence-informed interventions in a home setting. In addition, the HBHC program makes referrals and connects families to other services in the community as required.

### Evaluation of HBHC

In 2012–2013, MCYS introduced changes to the HBHC program to strengthen the existing program and provide better services to vulnerable families (MCYS, 2013). All health units across Ontario were required to implement these changes by April 2013. We were commissioned by MCYS to evaluate the new HBHC program over the first 6 months of implementation, and we used an embedded mixed-methods design for this evaluation (Creswell & Plano Clark, 2007). For the quantitative component, we analyzed client data from the provincial HBHC administrative database and administered online surveys to HBHC program staff in all health units involved in implementing the new HBHC program (i.e., the HBHC Health Unit Staff Survey). For the qualitative component, we conducted telephone focus groups with HBHC program staff to explore their perspectives and experiences in implementing the program changes. We chose the telephone focus group methodology because it allowed for HBHC staff across all health units in Ontario to participate within a short data collection time frame, without the extra cost and travel time for us and participants.

This evaluation received ethical approval from the Public Health Ontario Ethics Review Board (Study ID: 2013-012.01).

### Recruitment and Sampling

We recruited focus group participants in August 2013, through the HBHC Health Unit Staff Survey, which was sent to 1,128 staff. A description of the focus groups was included at the end of the online survey, and interested respondents were asked to provide their contact information using a separate web link to ensure confidentiality (i.e., contact information was not linked to survey responses).

Because staff with different roles in the program had different responsibilities and functions (Table 1), we conducted role-based focus groups, with each group consisting of participants in the same role. The purpose of this homogeneity as the basis of recruitment (Krueger & Casey, 2009) was to (a) understand the similarities or differences experienced by staff both within and between these roles, and (b) remove any power imbalance that may exist between these roles. Furthermore, inclusion of all roles gave an opportunity for staff who are normally less involved in decision making or program planning (e.g., family home visitors, data administrators) to provide their feedback on the program changes.

**Table 1.** Examples of Key Roles and Responsibilities of HBHC Program Staff.

HBHC Program Role	Examples of Key Roles and Responsibilities
HBHC program managers	Facilitate and coordinate HBHC programs in public health units.
	Manage resources, staff, and budgets.
	Participate in and collaborate with committees on family and reproductive health.
Public health nurses	Provide skills assessment to confirm risk of clients.
	Work with community partners to provide access to program and related information.
	Work collaboratively with the blended home visiting team and community partners to identify and negotiate family goals and update the family service plan.
	Identify the need for additional resources and supports, facilitate linkages to meet these needs over time.
Family home visitors	Support and facilitate parent-report-based screening.
	Support public health nurse assessment through observation of child and family needs and family interactions.
	Use role modeling to support skill and knowledge development during blended home visiting.
	Review and reinforce family goals.
	Assist families to access services.
Screening liaison nurses	Provide education and training related to population health, screening, health impacts of the early years, and risk factors to child development to community partners.
	Develop tools to support identification or quality completion of HBHC Screens.
	Create partnerships and collaboration to improve identification or HBHC Screen completion.
	Provide support for additional screening opportunities.
Data administrators	Perform data entry
	Generate reports using HBHC administrative database for monitoring purposes

*Note*. HBHC = Healthy Babies Healthy Children.

We used a combination of purposive sampling and random sampling to ensure broad representation across all health units for the five defined roles. The sampling procedure was conducted so that (a) all participants in each focus group were from different health units, (b) a maximum of two participants per health unit were selected for all focus groups, (c) roles with lower numbers of interested staff were selected first to ensure that they were given priority for participation, and (d) a maximum of 12 participants per focus group was not exceeded.

We e-mailed selected staff to invite them to participate in the focus group. In case they were unable to participate, another interested staff member with the same role in the same heath unit was invited. A reminder e-mail was sent to confirm participation 1 week before the focus group, with instructions on how to log into both the teleconference and webinar platform.

Participant consent was obtained in two time points. First, consent was obtained through their confirmation e-mail to participate. Second, electronic consent was obtained before the focus group discussion using the poll function of the webinar platform (this webinar function will be discussed in detail in the “Methodological Reflection” section of this article).

Five telephone focus groups were conducted in October 2013 (Table 2), and each focus group lasted about 2 hours. We developed an interview guide with semi-structured questions to guide the moderation of the focus group discussions. Three moderators took turns to moderate different questions throughout each focus group. After discussion, we gathered informal feedback from participants about this focus group format.

**Table 2.** Number of Interested, Invited, and Confirmed Participants in Each Focus Group.

Role-Based Focus Groups	Number of Interested Staff	Number of Invited Staff	Number of Participants
Data administrators	8	7	5
Screening liaison nurses	24	12	11
Family home visitors	27	12	11
Program managers	28	12	9
Public health nurses	63	12	10
Total number of staff and PHUs	150 staff from 35 PHUs	55 staff from 35 PHUs	46 staff from 31 PHUs

*Note*. PHUs =Public Health Units.

### Webinar Technology to Supplement Telephone Focus Groups

Given that the ability to generate interaction is an important characteristic differentiating focus groups from other data collection methods (Gothberg et al., 2013), it is crucial to ensure that telephone focus groups are able to maintain interactions between participants, especially in the absence of nonverbal cues. Therefore, we decided to add a webinar component to the telephone focus groups to augment the discussions and stimulate interactions. Although most webinar platforms have an audio conferencing function, which can eliminate the telephone dial-in, not all health unit staff have the required equipment to participate (e.g., no speaker/microphone). Therefore, we chose to use the regular phone instead of the audio conferencing function from the webinar platform.

We used Adobe® Connect™ as the webinar platform (Adobe Connect, 2014), which contains various features to enhance the interactions among focus group participants. In the subsequent sections, we share our process and reflect on our experiences of including this component to the telephone focus groups.

## Methodological Reflection

### How Did the Webinar Help Facilitate Telephone Focus Groups?

**Figure 1.**
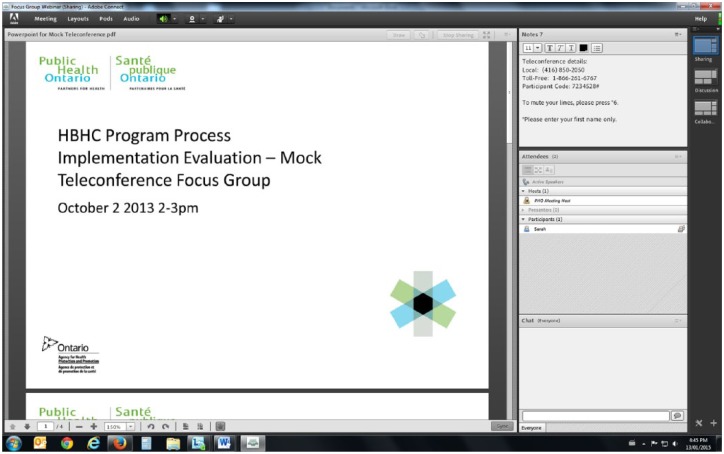
Screenshot of the Adobe® Connect™.

When participants logged into the webinar using the provided web link specifically created for the focus groups, they saw different display panels, or “pods,” on their computer screen (Figure 1). For example, participants’ and webinar hosts’ names were listed in the attendees pod, and the main display pod was used to share information posted by the moderators to guide the focus groups. To ensure participants remained as anonymous as possible, we asked participants to log into the webinar with their first name only and not to identify the health unit they worked in throughout the discussion.

As previously mentioned, participant consent was first obtained through their confirmation e-mail to participate. Before the focus group discussion, we asked participants to confirm their agreement to participate again by using the poll function. This function allowed us to create multiple choice questions to gather simple data from participants. We created a question, “I hereby confirm that I agree to participate in the focus group,” and asked them to choose an answer (Yes/No). The question popped up on participants’ screens, and they chose their answer accordingly. As the webinar host, we were able to see each participant and their answers. All participants confirmed their participation before the discussion began.

**Figure 2.**
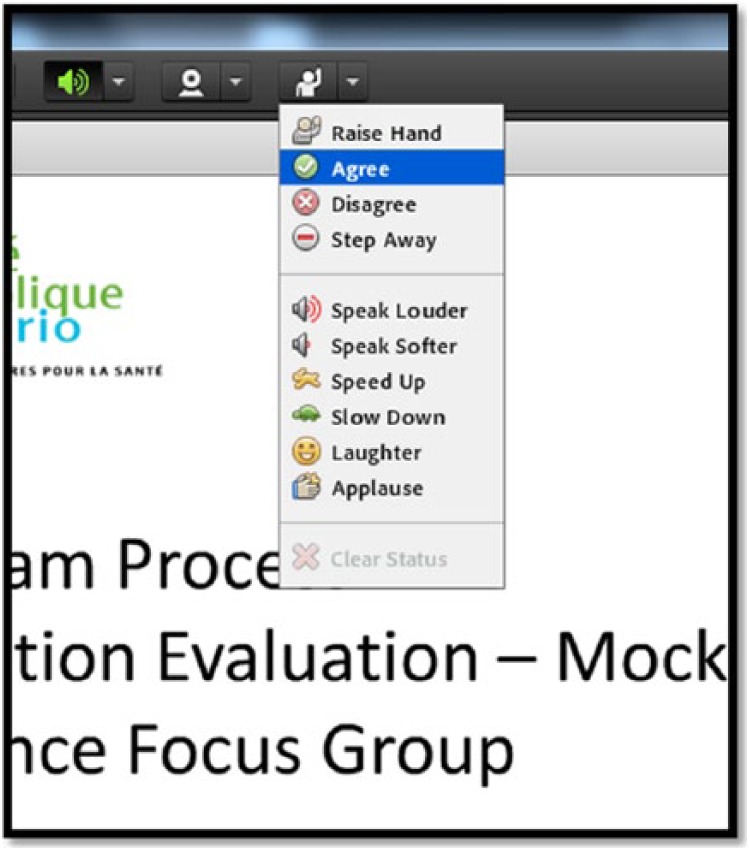
Screenshot of active participation features in Adobe® Connect™.

The active participation feature in Adobe Connect allowed participants to choose different statuses and provide visual feedback to all participants throughout the telephone focus groups. For our evaluation, the statuses most often used were as follows: “raise hand,” “agree,” and “disagree” (Figure 2). We instructed participants to use the “raise hand” status to inform us when they wanted to ask a question or join the discussion, the “agree” status to let others know that they agreed with the speaker or they would like to share similar comments, and the “disagree” status to indicate that their opinions or experiences differed from those of the speaker. Participants did not need to use this feature every time they wanted to speak; however, they could use it when another participant was speaking to ensure that only one person was speaking at a time for clarity purposes. Once a participant selected one of these statuses, the symbol remained next to their names until the moderator or they themselves selected the “clear status” option. This also allowed the moderators to give turns to participants who had something to add to the discussions.

We found that the active participation feature supported us to facilitate interactions between participants, as well as interactions between participants and moderators. In a face-to-face setting, participants would normally be able to express their wishes to speak through raising their hands or naturally joining the discussion after one person has finished speaking. The active participation features helped mimic this interaction, as both participants and moderators in the session were able to “see” those who wished to join the discussion through the webinar platform. Informal feedback gathered from participants after the focus groups also showed that they liked how the features can “visualize” their responses to the discussion, with comments such as, “I could do something to show that I was nodding my head.” In fact, a few of participants commented that this format (telephone with webinar) could be adapted for other purposes, such as training or virtual meetings. This indicates that participants have positive experience with this format in facilitating interactions and discussions in general.

Furthermore, these features allowed us to observe virtually the discussion and to ensure that participants had the opportunity to share their thoughts. For instance, when participants used the “disagree” status, we asked them to elaborate their thoughts to generate discussion. We found that the features facilitated the conversation flow and the focus groups ran smoothly.

Another webinar feature that helped us stimulate the interaction is the “Share My Screen” function, which allowed us to share the view of our desktop and applications with participants (Adobe Connect, 2014). We used this function to share a PowerPoint presentation with participants to guide the discussion with visuals and capture the discussion notes. Participants were able to see the note-taker’s screen through the webinar in real time and provided instantaneous feedback. In one instance, a participant informed us about a mistake in the discussion notes. This allowed us to mimic flipcharts used in a face-to-face environment, which was helpful to accurately capture participants’ opinions and encourage interaction between participants and moderators.

### What Were the Considerations or Challenges in Using Webinar to Facilitate Telephone Focus Groups?

To familiarize participants with the webinar features, it is necessary to allocate sufficient time demonstrating the webinar features, asking participants to practice the features from their computers (i.e., “chose the raise hand status”), and illustrating the icons that appeared beside their log-on names once they chose these features. For our evaluation, we allotted 15 minutes in the introduction solely to explain and practice the features. In several cases, participants did not find the features immediately during the practice exercise. To ensure that facilitating the focus groups ran smoothly, it is important that all participants were comfortable using these features. We also noticed that once participants learned how to use them, they used these features extensively throughout the discussion, which indicates that these features were useful for participants to express their thoughts as well. However, this means that the total amount of time required for telephone focus groups supplemented with webinar is longer than in face-to-face focus groups, and this is a logistical factor that needs to be considered.

As mentioned earlier, one of the identified advantages of telephone focus groups was that this approach can save time and money (Smith et al., 2009b). The experiences in our evaluation support this. We also felt that this approach was not too time-consuming to organize given that participants were located in a wide geographical area (i.e., it took 5 weeks from sending invitation e-mails to complete all focus groups). However, we found that facilitating telephone focus groups supplemented with webinar technology was more labor-intensive than moderating face-to-face focus groups. Traditional face-to-face focus groups usually have a moderator and an assistant moderator (Krueger & Casey, 2009). In our evaluation, we had three moderators and two assistants in each focus group. All of these roles proved to be important to ensure the focus groups went smoothly. In a face-to-face setting, one moderator would be able to facilitate the discussion while observing the visual cues simultaneously. In our case, one moderator was not able to simultaneously moderate the discussion and keep track of the icons that appeared beside the participants’ names that indicated their thoughts. Therefore, we had to divide the tasks, and we had to communicate among ourselves throughout the discussion to facilitate the focus groups smoothly (e.g., which participant’s turn to speak).

Despite explicit instructions on the specific technical requirements needed to use the webinar prior to the focus groups, at least one participant in each group experienced technical difficulties in the beginning of the session. This was mostly related to installing additional software on their computers to display the webinar platform and is beyond our control. Fortunately, the administrative staff and the IT staff in health units were able to resolve these issues quickly, and participants were able to successfully attend the focus groups. The availability of IT assistance at both the moderators’ and all participants’ ends is another factor to be considered. Another way to minimize this challenge in the actual focus group is to schedule a “trial run” prior to the actual focus group date. The purpose of this trial run is to test and make sure that all technical problems are resolved before the focus group. IT staff will be present at the practice run to discuss and assist with resolving any technical questions.

Although sound quality issues did not pose any limitations for any of the telephone focus groups, it is important for participants to have access to an environment with minimal distraction to participate fully in the discussion. Using a computer with an Internet connection throughout the focus group may itself provide a distraction (e.g., surfing Internet and checking e-mails). Also, because the focus group was scheduled during working hours, participants may experience interruptions from colleagues, and this could not be mitigated by the moderators.

Protecting participants’ privacy is a unique challenge for this approach. During one of the focus groups, we noticed that there was an uninvited individual logged in as “Trish” who was not on the invited list. When this individual was first noticed in the participant list, one of the moderators immediately sent a private message via the chat pod. However, this individual did not respond to the message. Before proceeding, all participants were asked via the telephone whether they had self-identified as “Trish” or whether they knew whether there was an individual “Trish” on the line. Again, we did not receive a response. We decided to proceed with the focus group as no participants expressed concern. During the subsequent focus groups, we took extra security measure by manually accepting every participant separately into the webinar. This measure worked well, and we did not experience similar incidents again. Researchers who wish to use this approach should carefully consider all measures to ensure the privacy of participants is protected to the best of their abilities. Furthermore, we could not fully ensure privacy for participants because the participants were responsible for self-selecting the appropriate environment in their individual setting. If a participant needed assistance from IT or colleagues at their health unit to participate, he or she needed to disclose his or her participation to others. Because each participant required access to a computer with Internet connection and a quiet private space to participate fully, these requirements may not be met by other target populations in other studies and may potentially create sampling bias.

Finally, although participants commented that the telephone focus groups with the webinar worked well overall, a few of participants mentioned that having visual cues could be beneficial to the discussion, and it allowed “you to put a face to a name.” Although the webinar features can support the interactions of participants in telephone focus groups, the availability of visual cues is critical in some cases that cannot be substituted by the webinar. Therefore, we advised that the decision to use the telephone focus group with the webinar approach should be driven not solely by the convenience of recruiting geographically dispersed participants or the lowered cost but also by the nature and the topic of the research or evaluation. In general, participants agreed that our approach worked well for the purpose and the topic of our evaluation.

## Discussion

To our knowledge, there is no literature to date on using webinar to augment the telephone focus groups. Our experience showed that the webinar is a beneficial addition to the telephone focus groups to simulate an interactive environment for participants. Similar to Wang and Hsu’s (2008) suggestion, we found that the webinar can mimic a face-to-face environment to facilitate a natural conversation among participants and to help with the flow of the discussion. The fact that participants suggested the use of teleconference and webinar for other purposes demonstrates that participants are receptive to this approach.

There are several limitations pertaining to this approach in our evaluation. First, this format worked well among this group of public health staff, who are familiar with teleconference discussions and use of webinar. However, this experience may not be generalizable to other target populations (i.e., those who are not familiar with webinars). Therefore, target populations should be taken into account when considering using telephone with webinars to collect focus group data.

In addition, although participants’ feedback and our experience indicated that the telephone focus group method worked well and the webinar functions stimulated an interactive environment, we did not conduct face-to-face focus groups in parallel. Therefore, we cannot know with certainty whether the topics discussed and participants’ interactions would compare with those in the traditional focus group format. There is limited literature that compares results between telephone and face-to-face focus groups, and the results are mixed (Frazier et al., 2010; Gothberg et al., 2013). Hence, more comparative qualitative research is needed in the future. Following Gothberg et al. (2013), we recommend that future studies focus on generating a better understanding of differences in participant interactions, breadth and depth of conversations, and adherence to the topic between different focus group formats.

## Conclusion

Based on our experience with the evaluation of the process implementation of the Ontario HBHC program, telephone focus groups with supplemental webinar technology offer a good alternative to face-to-face focus groups. Telephone focus groups facilitated data collection with participants, who are dispersed across wide geographical areas within a short time frame, and the webinar features effectively supported interaction among participants in telephone focus groups. As advances in technology continue to enhance our means of communication, researchers should continue to explore innovative ways to integrate technology and data collection for research and evaluation.
